# Cost analysis of the very elderly admitted to intensive care units

**DOI:** 10.1186/s13054-017-1689-y

**Published:** 2017-05-16

**Authors:** Nicolas Chin-Yee, Gianni D’Egidio, Kednapa Thavorn, Daren Heyland, Kwadwo Kyeremanteng

**Affiliations:** 1 Department of Medicine, University of Ottawa/The Ottawa Hospital, 501 Smyth Rd., Ottawa, Ontario K1H 8L6 Canada; 2 Ottawa Hospital Research Institute, The Ottawa Hospital, Ottawa, Ontario Canada; 3 Clinical Evaluation Research Unit, Kingston General Hospital, Kingston, Ontario Canada

**Keywords:** Critical illness, Cost of care, Octogenarian, End-of-life care, Outcome assessment

## Abstract

**Background:**

Very elderly patients are often admitted to intensive care units (ICUs) despite poor outcomes and frequent preference to avoid unnecessary prolongation of life. We sought to determine the cost of ICU admission for the very elderly and the factors influencing this cost.

**Methods:**

This prospective, observational cohort study included patients ≥80 years old admitted to 22 Canadian ICUs from 2009 to 2013. A subset of consenting individuals comprised a longitudinal cohort followed over 12 months. Costs were calculated from ICU length of stay and unit costs for ICU admission from a Canadian academic hospital. A generalized linear model was employed to identify cost-predictive variables.

**Results:**

In total, 1671 patients were included; 610 were enrolled in the longitudinal cohort. The average age was 85 years; median ICU length of stay was 4 days. Mortality was 35% (585/1671) in hospital and 41% (253/610) at 12 months. The average cost of ICU admission per patient was $31,679 ± 65,867. Estimated ICU costs were $48,744 per survivor to discharge and $61,783 per survivor at 1 year. For both decedents and survivors, preference for comfort measures over life support was an independent predictor for lower cost (*P* < 0.01).

**Conclusions:**

Considering the poor clinical outcomes, and that many ICU admissions may be undesired by very elderly patients, ICU costs in this population are substantial. Our finding that a preference for comfort care predicted a lower cost independent of mortality reinforces the importance of early goals of care discussions to avoid both undesired and potentially non-beneficial interventions, consequently reducing costs.

**Trial registration:**

ClinicalTrials.gov, NCT01293708. Registered on 10 February 2011.

**Electronic supplementary material:**

The online version of this article (doi:10.1186/s13054-017-1689-y) contains supplementary material, which is available to authorized users.

## Background

Very elderly (aged 80 or older) patients are being admitted to intensive care units (ICUs) in increasing numbers [1–3]. More concerning is that these admissions may occur despite the preference of many elderly patients to avoid unnecessary prolongation of life by life-sustaining therapy [4–6]. In spite of this, more than 70% of seriously ill hospitalized elderly patients do not discuss these preferences with their healthcare providers [6], which may result in provision of life-sustaining therapy even when patients prefer care focused on improved comfort and quality of life [7–10].

In our prospective, observational cohort of very elderly patients admitted to ICUs in Canada, not only was hospital and 12-month mortality high [11], but surviving individuals had a low probability of returning to baseline physical function [12]. Other studies have corroborated the findings of poor short- and long-term outcomes in this patient population [13–16]. These observations raised important questions about the appropriateness of both admission to and long-term treatment in the ICU for this population.

There is presently unprecedented strain on the healthcare system in the face of the baby boomer effect and increases in life expectancy [17], and critical care remains amongst the most expensive of healthcare interventions, consuming approximately 1% of the GDP [18, 19]. These costs continue to rise, and are expected to increase further as the incidence of critical illness requiring ICU admission is projected to increase by 80% by 2026 [19, 20]. There are significant economic implications surrounding admission of very elderly patients to the ICU, as the costs of providing prolonged and potentially non-beneficial care in this population are likely considerable. While ICU costs in elderly patients have been reported in smaller retrospective and prospective cohorts, estimates have ranged widely depending on the type of cost presented and the method of cost estimation [21–25].

The primary objective of this study was to determine contemporary costs of care for very elderly patients admitted to ICUs using our large prospective Canadian cohort. In addition, we sought to determine potentially predictive patient and family factors influencing cost of care.

## Methods

### Study design and population

As described in previous reports [11, 12], the data source for this study was a prospective cohort of patients 80 years of age or older admitted to 22 Canadian ICUs between September 2009 and January 2013. Briefly, all eligible patients whose hospital records were abstracted, based on research coordinator availability, were included in an unselected “hospital cohort”, which captured patient demographics and clinical outcomes for the index hospital admission. A subset of the hospital cohort from whom written informed consent was obtained (from patients and/or their primary caregivers) comprised a “longitudinal cohort” followed over 12 months, which described 12-month mortality and additional patient features, including family preferences for life-sustaining therapy. For this cohort, we excluded patients who remained in the ICU for less than 24 h, as well as non-residents of Canada and patients who did not have available family members or whose family members spoke neither English nor French. All those in the hospital cohort not fulfilling these exclusion criteria were approached for consent for the longitudinal study. Written informed consent was obtained from patients and/or their legal representatives before enrolment, and subsequently from competent, surviving patients for the follow-up assessment. Eligible family members (which included partners, significant others, and close friends) provided the most unpaid care to the patient, visited the patient at least once during ICU admission, and were at least 18 years of age. Local Research Ethics Board approval was obtained from all participating institutions.

### Outcome measures and explanatory variables

The primary outcome measure of this study was the ICU cost, which was calculated by multiplying total ICU length of stay for each patient by an average daily ICU cost per patient in 2012/13, derived using the case-costing system within the Ottawa Hospital Data Warehouse. This case-costing system estimates total daily direct and indirect costs by taking the sum of costs of each functional centre, such as medications and laboratory tests, incurred by each patient for each ICU day. The system is based on a standardized costing methodology developed by the Ontario Case Costing Initiative according to the Canadian Institute for Health Information Management Information Systems guidelines [26]. The average daily ICU cost per patient was obtained by dividing total ICU costs incurred between April 2012 and March 2013 by the total number of ICU patient days during the same period.

To estimate adjusted hospital costs, we included the following variables in our regression analyses: age, sex, primary ICU admitting diagnosis, admission type (medical, surgical elective, surgical emergency), Charlson comorbidity index [27], Acute Physiology and Chronic Health Evaluation (APACHE) II score [28], and Sequential Organ Failure Assessment (SOFA) score at admission [29]. Additional variables studied for the longitudinal cohort included patient frailty (as per the Rockwood Clinical Frailty Score [30], where a score of >4/7 was considered frail), residence in a nursing home, family preference for life-sustaining therapy, and presence of an advance directive. As explained in greater detail in the initial report [11], for family preference for life-sustaining therapy, patients’ family members were asked to choose between life support, comfort measures without life support, and “I am unsure”. Only the presence of an advance directive was recorded; contents of these directives were not captured.

### Statistical analyses

Cost of ICU was expressed as the mean (± standard deviation) of ICU admission per patient in Canadian Dollars. We estimated ICU cost per survivor to discharge and per survivor at 1 year from total ICU costs and mortality in the hospital and longitudinal cohorts, respectively. A generalized multivariable linear regression model using a log link function and gamma distribution was employed to predict ICU costs while controlling for other factors. This regression was performed separately for survivors and hospital decedents. Results were expressed as standardized (beta) coefficients with 95% confidence intervals (CIs). *P* values less than 0.05 were considered statistically significant. All analyses were performed using SAS (Version 9.3, Cary, NC, USA).

## Results

### Patient characteristics and clinical outcomes

Of 3064 patients aged 80 years or older admitted to ICUs who were screened for eligibility, 1671 had hospital records abstracted and were included in the hospital cohort; 610 of these consented to be enrolled in the longitudinal cohort. Table 1 summarizes the baseline characteristics and clinical outcomes of patients aged 80 years or older admitted to ICUs for both the hospital and longitudinal cohorts, as described in previous reports [11, 12]. In the hospital cohort, median ICU length of stay was 4 days and 585 patients (35%) died in hospital. In the longitudinal cohort, median ICU length of stay was 6 days, 158 patients (26%) died in hospital, and 253 (41%) were deceased within 12 months. With respect to family preferences for life-sustaining treatment, 310 (51%) preferred life support, 129 (21%) desired comfort care without life support, and 171 (28%) were unsure or had not specified their preference. Three-hundred (49%) patients had an advance directive.

**Table 1:** Baseline characteristics and clinical outcomes of study patients

Characteristic/outcome	Hospital cohort (*n* = 1671)	Longitudinal cohort (*n* = 610)
Age (years)	85 ± 3	84 ± 3
Sex (male)	915 (55%)	338 (55%)
Admission APACHE II score	22 ± 8	22 ± 7
Baseline SOFA score	5 ± 3	5 ± 3
Charlson comorbidity index	2 ± 2	2 ± 2
Admission type		
Medical	1,033 (62%)	377 (62%)
Surgical elective	220 (13%)	83 (14%)
Surgical emergency	418 (25%)	150 (25%)
Primary ICU admitting diagnosis		
Cardiovascular	408 (24%)	143 (23%)
Respiratory	389 (23%)	157 (26%)
Gastrointestinal	298 (18%)	110 (18%)
Sepsis	178 (11%)	72 (12%)
Other	398 (24%)	128 (21%)
Frailty index >4/7	N/A	193 (32%)
Residence in a nursing home	N/A	24 (4%)
Family preference for life-sustaining treatment		
Life support	N/A	310 (51%)
Comfort care without life support	N/A	129 (21%)
Unsure/unclear/missing	N/A	171 (28%)
Presence of advance directive	N/A	300 (49%)
Median ICU length of stay	4 (2–8)	6 (3–10)
Median hospital length of stay	17 (8–33)	21 (12–40)
Mean ICU length of stay	9 ± 19	8 ± 9
Mean hospital length of stay	9 ± 21	11 ± 14
Hospital mortality	585 (35%)	158 (26%)
12-month mortality	N/A	253 (41%)

Values are shown as mean ± standard deviation, median (interquartile range), or count (%). *APACHE* Acute Physiology and Chronic Health Evaluation, *ICU* intensive care unit, *N/A* not applicable, *SOFA* Sequential Organ Failure Assessment score

### Cost of ICU admission

Table 2 illustrates separately the cost of ICU admission for survivors and decedents in both cohorts. The average costs of ICU admission per patient were $31,679 ± 65,867 and $36,158 ± 34,222 for the hospital and longitudinal cohorts, respectively. For the hospital cohort, the cost of ICU in survivors was $27,833 ± 46,359 while the cost in non-survivors was $38,820 ± 91,294.

**Table 2:** Cost of ICU admission for very elderly patients

Group	*n*	Cost of ICU admission per patient	
		Mean	Standard deviation
Hospital cohort			
All patients	1,671	31,679	65,867
All patients, per day	1,671	3520	7749
Survivors to discharge	1,086	27,833	46,359
Decedents in hospital	585	38,820	91,294
Longitudinal cohort			
All patients	610	36,158	34,222
All patients, per day	610	4520	3845
Survivors to discharge	452	31,323	31,228
Decedents in hospital	158	49,989	38,491

Costs are presented in Canadian Dollars. Costs of ICU admission were calculated by ICU length of stay and both indirect and variable direct costs for ICU admission at The Ottawa Hospital in 2012–2013. *ICU* intensive care unit, *SD* standard deviation

**Fig. 1:**
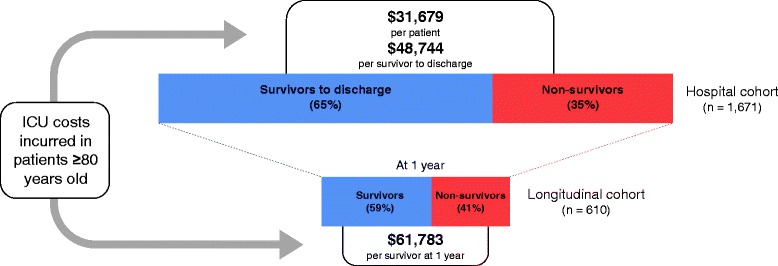
Intensive care unit (*ICU*) costs in very elderly patients with respect to mortality. Average cost of ICU admission per patient (hospital cohort) and calculated total ICU cost per survivor to discharge and at 1 year. The longitudinal cohort was a selected cohort of individuals from the hospital cohort followed over a 1-year period. Costs are presented in Canadian Dollars

With respect to outcomes, the estimated total ICU cost was $48,744 per survivor to discharge (hospital cohort) and $61,783 per survivor at 1 year (longitudinal cohort) (Fig. 1). This estimation does not include costs of hospitalization following ICU discharge.

### Predictive variables

Table 3 demonstrates the multivariate linear regression results for predictive variables for cost of ICU admission performed separately for survivors to discharge and decedents in hospital. For survivors and decedents, respectively, older age was associated with lower cost (coefficient = −0.139, 95% CI −0.021 to −0.007, *P* = 0.039; coefficient = −0.029, 95% CI −0.051 to −0.007, *P* = 0.008) and respiratory primary ICU diagnosis was associated with a greater cost (compared with cardiovascular diagnosis; coefficient = 0.461, 95% CI 0.294 to 0.627, *P* = 0.000; coefficient = 0.690, 95% CI 0.270 to 1.111, *P* = 0.001). Amongst patients who died in hospital, a greater baseline SOFA score, but not greater comorbidity index or APACHE II score, was associated with lower cost (coefficient = −0.084, 95% CI −0.114 to −0.053, *P* = 0.000). For survivors, increased comorbidity index was a predictor for lower cost (coefficient = −0.035, 95% CI −0.069 to −0.000, *P* = 0.048), and higher APACHE II score was a predictor for greater cost (coefficient = 0.031, 95% CI 0.023 to 0.039, *P* = 0.000); SOFA score was not significantly associated with the cost of ICU care in this group.

**Table 3:** Multivariate model for predictors of ICU cost in very elderly patients

Variable	Coefficient (95% CI)	*P* value	Coefficient (95% CI)	*P* value
Hospital cohort (*n* = 1671)	Survivors to discharge (*n* = 1,086)		Decedents in hospital (*n* = 585)	
Age (older)	−0.139 (−0.021, −0.007)	0.039	−0.029 (−0.051, −0.007)	0.008
Sex (female)	−0.092 (−0.207, 0.022)	0.113	−0.034 (−0.289, 0.221)	0.796
Admission APACHE II score (greater)	0.031 (0.023, 0.039)	0.000	0.001 (−0.012, 0.014)	0.926
Baseline SOFA score (greater)	−0.005 (−0.028, 0.018)	0.661	−0.084 (−0.114, −0.053)	0.000
Charlson comorbidity index (greater)	−0.035 (−0.069, −0.000)	0.048	0.024 (−0.019, 0.067)	0.281
Admission type				
Surgical elective vs. medical	−0.133 (−0.287, 0.021)	0.091	0.193 (−0.191, 0.577)	0.324
Surgical emergency vs. medical	0.076 (−0.065, 0.218)	0.290	0.091 (−0.169, 0.351)	0.494
Primary ICU diagnosis				
Respiratory vs. cardiovascular	0.461 (0.294, 0.627)	0.000	0.690 (0.270, 1.111)	0.001
Gastrointestinal vs. cardiovascular	0.150 (0.004, 0.295)	0.043	0.277 (−0.088, 0.642)	0.137
Sepsis vs. cardiovascular	0.177 (−0.029, 0.382)	0.092	0.538 (0.238, 0.839)	0.000
Other vs. cardiovascular	0.174 (0.020, 0.328)	0.027	−0.210 (−0.463, 0.042)	0.103
Longitudinal cohort (*n* = 610)	Survivors to discharge (*n* = 452)		Decedents in hospital (*n* = 158)	
Frailty index >4/7	−0.001 (−0.071, 0.069)	0.977	0.044 (−0.044, 0.132)	0.325
Residence in a nursing home	−0.321 (−0.633, −0.010)	0.043	0.046 (−0.485, 0.577)	0.864
Family preference for life-sustaining treatment				
Comfort care vs. life support	−0.253 (−0.433, −0.073)	0.006	−0.402 (−0.650, −0.155)	0.001
Unsure/unclear/missing vs. life support	−0.429 (−0.635, −0.223)	0.000	−0.393 (−0.814, 0.028)	0.067
Presence of advance directive	0.122 (−0.050, 0.294)	0.165	0.214 (−0.010, 0.438)	0.061

Generalized linear model using a log function. Cost distribution closely matched gamma distribution (incorporated into model). Regression was performed separately for each cohort, and for both survivors and decedents. See Additional file 2 (Table S1) for the complete regression results for the longitudinal cohort. *APACHE II* Acute Physiology and Chronic Health Evaluation score, *CI* confidence interval, *ICU* intensive care unit, *SOFA* Sequential Organ Failure Assessment score

Compared with patients whose families preferred life support, those with a family member specifying a preference for comfort care without life support had a significantly lower ICU cost in both survivors and decedents (coefficient = −0.253, 95% CI −0.433 to −0.073, *P* = 0.006; coefficient = −0.402, 95% CI −0.650 to −0.155, *P* = 0.001, respectively). Neither clinical frailty nor the presence of an advance directive were significantly associated with the cost of ICU care. Although residence in a nursing home was associated with a lower cost of care amongst survivors (coefficient = −0.321, 95% CI −0.633 to −0.010, *P* = 0.043), it was not a significant cost predictor in those who died in hospital.

## Discussion

In this multicentre cohort study, we demonstrated the significant cost of ICU admission in very elderly patients—approximately $32,000 per patient. Despite this cost, clinical outcomes were poor: 35% of patients died in hospital and 41% of those followed longitudinally were deceased at 1 year. This amounts to an ICU cost alone of nearly $49,000 per survivor to discharge for the unselected cohort, and $62,000 per survivor at 1 year amongst those in the longitudinal study. In addition, we demonstrated important patient and family factors that influence the cost of care; specifically, a preference for comfort measures over life support was an independent predictor for lower cost of care, and remained a predictor for both survivors and decedents.

Our reported cost of ICU admission in very elderly Canadians of $31,679 per patient is considerably higher than most studies reporting the cost of ICU care in similar populations [21–25]. Interpretation of cost differences between studies, however, remains inaccurate and may be misleading given the various types of costs reported and differences in methods of cost calculation. As a result, previous studies that calculated the cost of ICU admission in elderly and very elderly patients report a wide range of costs, from approximately $3300 to $28,100 USD per patient [21–25]. The daily ICU cost per patient in the present study, however, was similar compared with reported figures from cohorts that also included non-elderly adult patients [18, 25, 31]. For Canadian adults, a recent report estimated an average daily ICU cost of $3592 with an average length of stay in the ICU of 3 days [32]. Altogether, the similar daily cost but longer total length of stay in the ICU for the very elderly patients in our cohort compared to the general adult ICU population likely accounts for the notably higher overall cost of ICU admission [31, 32]. Although some studies suggest that very elderly patients admitted to ICU may incur fewer costs compared to their younger counterparts as a result of less aggressive care, our study did not directly compare costs to younger patient cohorts or study the costs associated with specific interventions [21, 22]. This trend towards fewer invasive interventions in very elderly patients, however, was not observed in our cohort, 85% of whom received at least one form of life-sustaining therapy and 72% of whom were mechanically ventilated [11], contrasting with the estimated rate of mechanical ventilation in the general adult ICU population in Canada of 33% [32]. This might, in fact, suggest that average daily ICU costs may underestimate costs for elderly patients and overestimate costs for younger adults.

The cost of ICU stay for the very elderly becomes more striking when contemplated in light of clinical outcomes observed in this population. Without even considering the costs of non-ICU hospitalization or readmission, long-term care, and outpatient care, the cost of ICU alone in our study was over $60,000 per survivor at 1 year. Though 1-year mortality was greater than 40% in our study, other investigators have reported that long-term mortality may be even higher in this group (55–90% at 3 years) [15, 16, 33]. Furthermore, we recently showed, in the same cohort as the present study, that at 1 year only 26% of patients had survived and recovered back to, or near, their baseline level of functioning [12]. As such, the ICU and non-ICU costs per functional survivor are likely substantially higher than the exclusive ICU costs we present. Potential explanations for the differences in mortality and length of stay between the hospital and longitudinal cohorts are discussed in greater detail in earlier reports [11, 12].

Even more important than the clinical outcomes were the apparent incongruences between patient/family wishes and the provision of life-sustaining treatment received, which many elderly patients would have preferred to avoid [4–6]. Nearly one quarter of patients or their caregivers in the present study had an expressed preference for comfort measures over life support, yet were admitted to ICU and received life-sustaining therapy. These observations should prompt healthcare providers to re-evaluate the benefit of ICU care for the very elderly, and suggest that patients’ preferences and goals of care are not addressed early enough to prevent unwanted healthcare interventions or ICU admissions.

Mechanical ventilation has been shown to increase ICU costs [31]. This may partially explain why respiratory diagnoses in our cohort were associated with the highest costs in our study. The observation that cardiovascular diagnoses were the least costly probably reflects the inclusion of post-operative cardiac and vascular surgery patients in this group—patients who may have more predictable ICU courses, preselected based on fitness for surgery. Perhaps not surprisingly, older age and greater SOFA scores in non-survivors were associated with a lower cost, likely because they were predictors of earlier mortality. In survivors to discharge, why older age was a predictor of lower cost and why we observed conflicting results with respect to comorbidity index and illness severity scores as cost-predictive factors remains unclear.

Interestingly, we found that patients whose family members had specified a preference for comfort care over life support had a significantly lower cost of ICU care, not only for those who died in hospital, but also for survivors to discharge. This finding suggests that, without adversely affecting clinical outcomes, an approach that focuses early on comfort measures instead of life support led to a reduction in cost. This is corroborated by our recent work which demonstrated that palliative care consultation reduced ICU length of stay [34].

The main limitations to our study surrounded the calculation of ICU cost. Our cost estimates were based on figures from a single academic institution, and therefore do not account for variability in costs between institutions and the known lower costs in the community ICU setting [32]. Additionally, costs were estimated by ICU dates of admission (length of stay), and therefore specific costs of care for each patient were not captured. These are known limitations to this validated method of cost calculation [35]. Finally, inpatient and outpatient costs of care following ICU discharge were not ascertained, and would be an important consideration as many of these patients have extended hospitalizations and a prolonged recovery period.

## Conclusions

Our large multicentre cohort provides a unique and representative population to define the cost of critical care for the very elderly, which is higher than in the general adult ICU population despite more than double the ICU mortality rate [32]. Considering that very elderly patients frequently express a desire for a good quality of life over life-sustaining therapy, our finding that a family preference for comfort care was a predictor for lower ICU cost provides further impetus for early goals of care discussion and the development of tools for recognition of patients with probable poor outcomes [36]. Our reported cost figures will be important to inform future clinical, policymaking, and funding decisions aiming to reduce financial strain on our medical system and ensure appropriate allocation of healthcare resources. Ultimately, this will require systemic changes in end-of-life care, specifically surrounding the decisions to continue life-sustaining therapy for an elderly patient with multiple comorbidities in the ICU.

## Additional files

Additional file 1: List of participating centres. (DOCX 37 kb)

Additional file 2: Table S1. Multivariate model of ICU cost in very elderly patients: longitudinal cohort (*n* = 610). (DOCX 13 kb)

## Abbreviations

APACHE II: Acute Physiology and Chronic Health Evaluation II
CI: Confidence interval
ICU: Intensive care unit
SOFA: Sequential Organ Failure Assessment
