# Supplementary material for: Cost analysis of the very elderly admitted to intensive care units
Source: Crit Care. 2017 May 16;21:109. doi: 10.1186/s13054-017-1689-y (PMC5433056; doi:10.1186/s13054-017-1689-y)
Supplement: Supplementary file 1 — List of participating centres. (DOCX 37 kb) [file 13054_2017_1689_MOESM1_ESM.docx]

**Supplemental file 1. List of participating centres**

Participating Sites (number in brackets refers to number of patients enrolled in longitudinal study at each site).

Hôpital du Sacré-Coeur de Montréal (71), Montréal, Québec

Hôpital Enfant-Jesus (50), Quebec City, Quebec

Hôpital Laval, (50), Quebec City, Quebec

Royal University Hospital (49), Saskatoon, Saskatchewan

University of Alberta Hospital (48): Edmonton, Alberta

St. Paul’s Hospital (46), Vancouver, British Columbia

Kingston General Hospital (39), Kingston, Ontario

Ottawa Hospital, Civic Campus (32), Ottawa, Ontario

Royal Alexandra Hospital (31), Edmonton, Alberta

Foothills Hospital, Calgary, Alberta (29)

St. Boniface Hospital Winnipeg (29), Winnipeg, Manitoba

Hôpital Maisonneuve-Rosemont (24), Montreal, Quebec

Mount Sinai Hospital (19), Toronto, Ontario

Royal Columbian (17), New Westminster, BC

Sunnybrook Health Sciences Centre (17), Toronto, Ontario

St. Michael’s Hospital MSICU/NTICU(14/0), Toronto, Ontario

Peter Lougheed Hospital (11) Calgary, Alberta

Toronto General Hospital (11), Toronto, Ontario

St. Joseph’s Healthcare (11), Hamilton, Ontario

Victoria General Hospital (5), Victoria, British Columbia

Royal Jubilee Hospital (5), Victoria, British Columbia

Winnipeg Health Sciences Centre (2), Winnipeg, Manitoba
