# Supplementary material for: Cost analysis of the very elderly admitted to intensive care units
Source: Crit Care. 2017 May 16;21:109. doi: 10.1186/s13054-017-1689-y (PMC5433056; doi:10.1186/s13054-017-1689-y)
Supplement: Supplementary file 2 — Multivariate model of ICU cost in very elderly patients: longitudinal cohort (n = 610). (DOCX 13 kb) [file 13054_2017_1689_MOESM2_ESM.docx]

**Table S1. Multivariate model of ICU cost in very elderly patients: longitudinal cohort (n=610)**

| **Variable** | **Survivors to discharge (n=452)** | |  | **Decedents in hospital (n=158)** | |
| --- | --- | --- | --- | --- | --- |
|  | **Coefficient (95% CI)** | **P value** |  | **Coefficient (95% CI)** | **P value** |
| Age (older) | -0.015 (-0.034, 0.005) | 0.143 |  | -0.059 (-0.088, -0.030) | 0.000 |
| Sex (female) | -0.023 (-0.224, 0.178) | 0.820 |  | -0.074 (-0.380, 0.232) | 0.637 |
| Admission APACHE II score | 0.024 (0.013, 0.036) | 0.000 |  | -0.003 (-0.021, 0.015) | 0.752 |
| Baseline SOFA score | 0.040 (-0.006, 0.085) | 0.087 |  | -0.103 (-0.147, -0.059) | 0.000 |
| Charlson comorbidity index (greater) | -0.071 (-0.120, -0.022) | 0.004 |  | -0.009 (-0.050, 0.032) | 0.660 |
| Frailty index >4/7 | -0.001 (-0.071, 0.069) | 0.977 |  | 0.044 (-0.044, 0.132) | 0.325 |
| Residence in a nursing home | -0.321 (-0.633,-0.010) | 0.043 |  | 0.046 (-0.485, 0.577) | 0.864 |
| Admission type |  |  |  |  |  |
| Surgical Elective | -0.220 (-0.437, -0.002) | 0.048 |  | 0.015 (-0.518, 0.547) | 0.957 |
| Surgical Emergency | 0.103 (-0.116, 0.322) | 0.357 |  | -0.499 (-0.851, -0.146) | 0.006 |
| Primary ICU Diagnosis |  |  |  |  |  |
| Respiratory vs. cardiovascular | 0.452 (0.198, 0.707) | 0.000 |  | -0.143 (-0.577, 0.292) | 0.521 |
| Gastrointestinal vs. cardiovascular | 0.111 (-0.126, 0.348) | 0.359 |  | 0.367 (-0.041, 0.776) | 0.078 |
| Sepsis vs. cardiovascular | 0.149 (-0.117, 0.414) | 0.272 |  | 0.093 (-0.331, 0.517) | 0.668 |
| Other vs. cardiovascular | 0.348 (0.083, 0.613) | 0.010 |  | -0.485 (-0.937, -0.034) | 0.035 |
| Family preference for life-sustaining treatment |  |  |  |  |  |
| Comfort care vs. life support | -0.253 (-0.433, -0.073) | 0.006 |  | -0.402 (-0.650, -0.155) | 0.001 |
| Unsure/unclear/missing vs. life support | -0.429 (-0.635, -0.223) | 0.000 |  | -0.393 (-0.814, 0.028) | 0.067 |
| Presence of advance directive | 0.122 (-0.050, 0.294) | 0.165 |  | 0.214 (-0.010, 0.438) | 0.061 |

**Legend:** Generalized linear model using a log function. Cost distribution closely matched gamma distribution (incorporated into model). CI: confidence interval; APACHE II: Acute Physiology and Chronic Health Evaluation score; SOFA: Sequential Organ Failure Assessment score.
